# Case Report: Inferior Vena Cava Agenesia in a Young Male Patient Presenting With Bilateral Iliac Veins Thrombosis

**DOI:** 10.3389/fsurg.2022.832336

**Published:** 2022-03-22

**Authors:** Edoardo Pasqui, Gianmarco de Donato, Silvia Camarri, Raffaele Molinari, Irene Cascinelli, Veronica Pelini, Luigi Abate, Giancarlo Palasciano

**Affiliations:** ^1^Department of Vascular Surgery, University of Siena, Siena, Italy; ^2^Division of Internal Medicine, Associated Hospitals in Val di Chiana, Montepulciano, Italy; ^3^Division of Radiology, Associated Hospitals in Val di Chiana, Montepulciano, Italy

**Keywords:** inferior vena cava, agenesia, deep vein thrombosis, venous thromboembolism (VTE), anticoagulants

## Abstract

**Introduction:**

Anomalies in inferior vena cava represent an uncommon finding with a prevalence of 0. 3 to 0.5% among healthy patients. Specifically, the condition characterized by the agenesis of the inferior vena cava (IVC; AIVC) has been observed among the 0.0005 to 1% of the general population. AIVC is strongly related to deep vein thrombosis (DVT) of the lower limb and pelvic district, especially in young patients. The rarity of the presented condition could relate to an underestimation of its impact on a particular clinical setting leading to a delayed diagnosis and inaccurate early- and long-term management.

**Report:**

We presented a case of this anomaly regarding a 31-year-old man presenting with bilateral symptomatic proximal DVT. Duplex vascular ultrasound and subsequent CT-angiography revealed the complete occlusion of the right external and common iliac vein, as well as partial occlusion of the contralateral external iliac vein, in the patient. The exam also revealed the interruption of IVC in its infrarenal part. At the level of renal veins coalescence, IVC appeared again in its usual position. A dilatated portal system, hepatic veins, and azygos and hemiazygos systems were also highlighted. Anticoagulation was promptly started with the administration of Fondaparinux (7.5 mg/die). In addition, compression stocking was initiated within 24 h from diagnosis. After 3 weeks, the anticoagulation regimen was shifted toward the administration of a direct oral anticoagulant (Apixaban; 5 mg two times a day). At 1-month follow-up, a vascular duplex ultrasound revealed a complete resolution of the iliac veins' thrombosis.

**Conclusion:**

It is important to consider the eventuality of IVC anomalies in a young adult presenting with unexplained, extensive, or bilateral DVT. Accurate diagnostic evaluation is necessary to fully identify this condition that could represent a real challenge.

## Introduction

Anomalies in inferior vena cava (IVC) represent an uncommon finding. Previous studies reported a prevalence of 0.3 to 0.5% of healthy patients. Specifically, the condition characterized by the agenesis of the IVC (AIVC) has been observed among the 0.0005–1% of the general population (1). AIVC is strongly related to deep vein thrombosis (DVT) of the lower limb and pelvic district, especially in young patients.

The rarity of the presented condition could relate to an underestimation of its impact on a particular clinical setting leading to a delayed diagnosis and inaccurate early- and long-term management. We present a case of this anomaly regarding a male patient presenting with bilateral symptomatic proximal DVT.

Full written informed consent from the patient was obtained for publishing this article and images.

Ethical approval was not necessary for this case.

## Case Report

A patient who is a 31-year-old man with no previous history of arterial or venous thrombotic events was admitted to the emergency department with bilateral lower limb swelling, majorly located at the left lower limb. Clinical history was mute with no drugs assumption. The patient was a Non-smoker and did not assume alcohol. The patient has a normal weight and is physically active. Familiar anamnesis was mute for venous thromboembolic events. Physical examination highlighted a bilateral swelling of lower limbs. Abdominal superficial veins were not dilatated and no sign of varicocele was present. The patient referred that symptomatology occurred a week before with a gradual worsening and cranial extension. Bauer and Homans maneuvers were slightly positive. Venous duplex ultrasound was performed, revealing a marked reduction of physiological flow modulation at the level of femoral veins bilaterally with no presence of hypoechoic material within. Abdominal large veins examination highlighted a significant bilateral dilatation of iliac veins with the presence of hypoechoic material, suggesting the diagnosis of a proximal DVT. Laboratory tests revealed a mild increase of inflammatory markers and a significant increase of fibrinogen (756 mg/dl) and D-Dimer level (>5,000 ng/ml).

**Figure 1:**
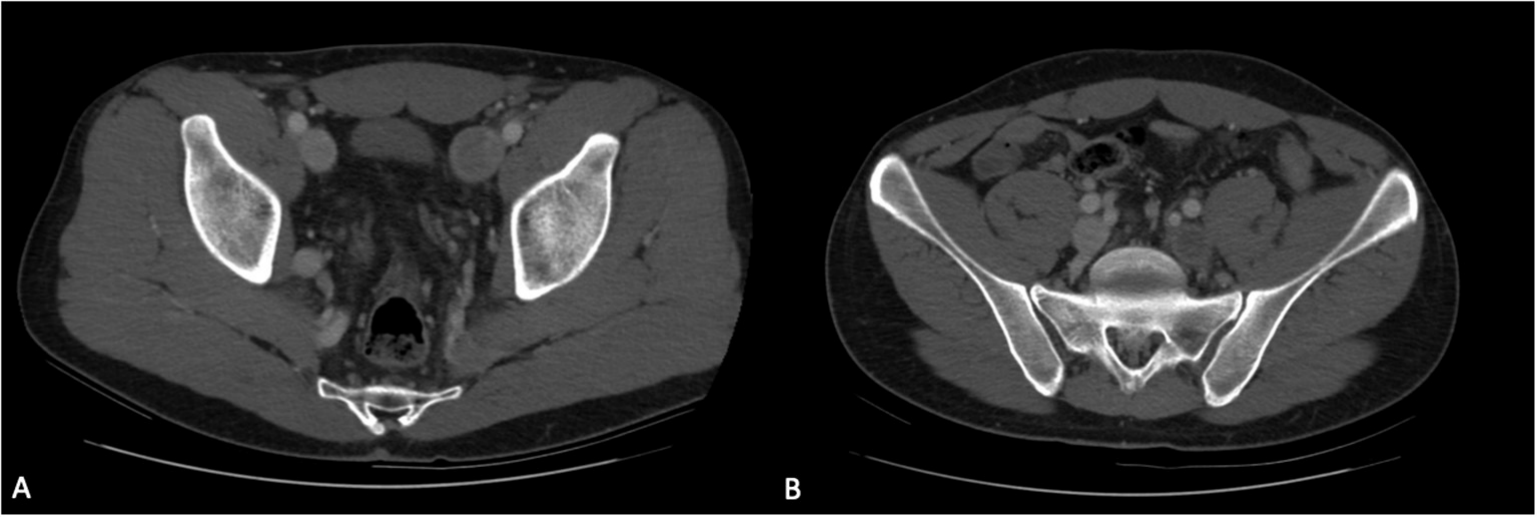
**(A,B)** Patient's CT-angiography at admission revealing complete thrombosis of left external iliac vein, right internal iliac vein, and partial thrombosis of right external iliac vein.

**Figure 2:**
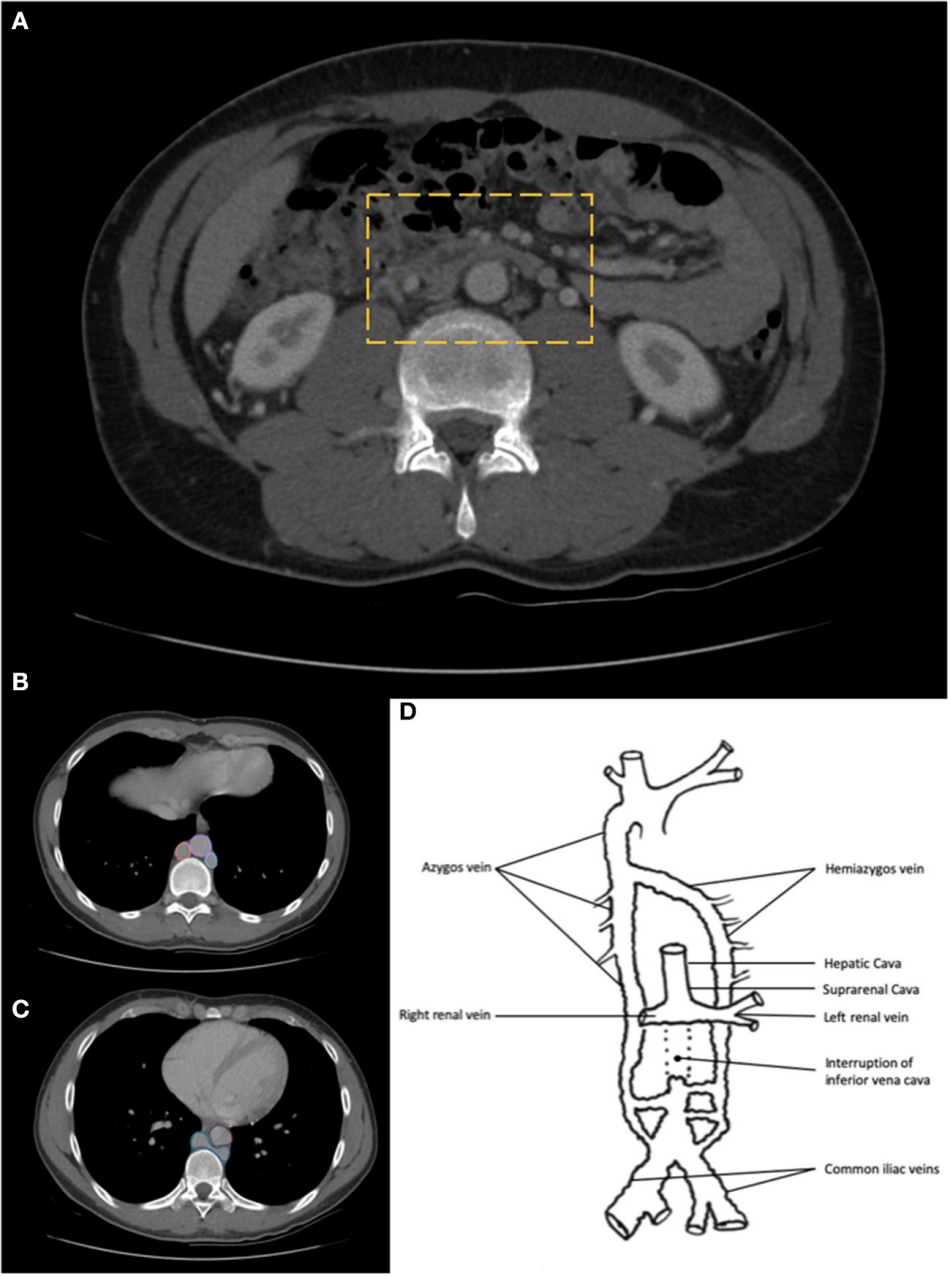
**(A)** CT-angiography reveals the complete absence of the infrarenal part of inferior vena cava (IVC). **(B)** Thorax CT-angiography highlights the role of the dilatation of azygos and hemiazygos systems. (Pink circle: Azygos vein; Violet circle: Aorta; Blue circle: Hemiazygos vein); **(C)** Thorax CT-angiography slice indicates the level of azygos and hemiazygos coalescence (blue shape). Aorta is highlighted by a purple circle; **(D)** Depicting the scheme of the anatomical patient's variant, highlighting the IVC agenesia and the development of the collateral venous drainage systems.

The patients underwent a CT-angiography that confirmed the presence of a bilateral iliac DVT, with complete occlusion of the right external and common iliac vein and partial occlusion of the contralateral external iliac vein (Figures 1A,B). The exam also revealed the interruption of IVC in its infrarenal part (Figure 2A). At the level of renal veins coalescence, IVC appeared again in its usual position. A dilatated portal system and hepatic veins were also highlighted. From the abdomen toward the thorax, azygos and hemiazygos systems became more and more evident. After the coalescence with hemiazygos, the azygos vein significantly reached a diameter comparable to the aorta (22 mm) (Figures 2B,C).

No evidence of pulmonary embolism was outlined.

Furthermore, a diagnosis of infrarenal AIVC was made (Figure 2D). Anticoagulation was promptly started with the administration of Fondaparinux (7.5 mg/die). In addition, compression stocking was initiated within 24 h from diagnosis. Screening for thrombophilia was also performed, wherein an activated protein C resistance emerged.

The patient was discharged after 4 days of hospitalization uneventfully. After 3 weeks, the anticoagulation regimen was shifted toward the administration of a direct oral anticoagulant (Apixaban; 5 mg two times a day). Vascular duplex ultrasound was repeated at 1- and 3-months follow-up with a complete resolution of the iliac veins' thrombosis. Anticoagulation continued for at least 12 months in order to avoid early recurrence.

## Discussion

**Figure 3:**
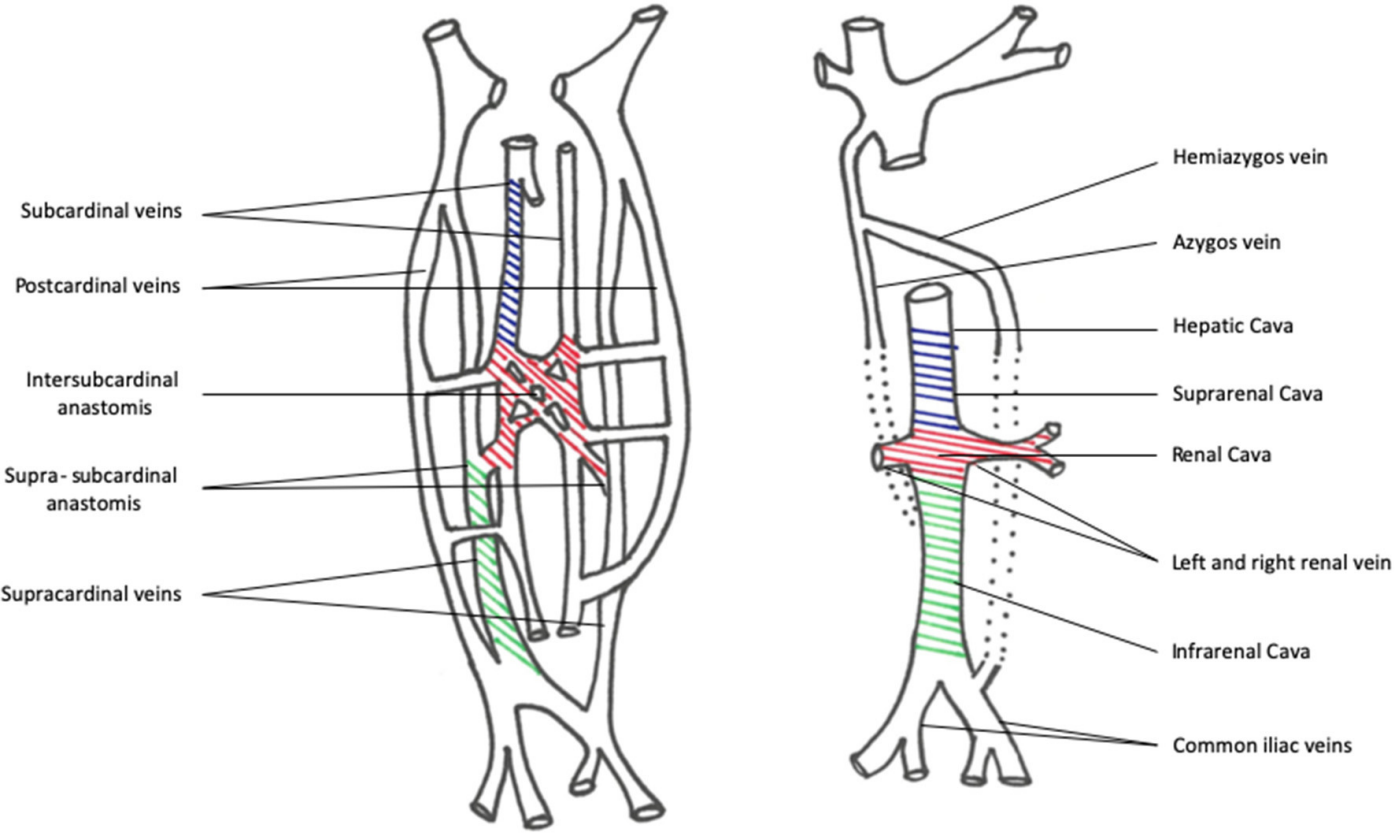
Picture of embryological development pathway of the IVC system. On the left, the schematic configuration highlights the embryological precursors of the IVC: blue indicated the subcardinal veins system; red indicates the Intersubcardinal network anastomosis; green indicates the supracardinal veins. After a complex development and regression process, they give origin to the final suprarenal cava, renal cava, and infrarenal cava.

The embryological pathway of IVC development is quite complex (Figure 3). Three paired venous networks provide the basis of IVC in the very first weeks of embryonic life. The posterior cardinal, subcardinal, and supracardinal veins develop consecutively. In the end, the orthotopic IVC (right-sided) reaches its final configuration when some of these venous networks anastomose and others regress. The suprarenal IVC originates from the right subcardinal vein, while the infrarenal IVC forms from the right supracardinal vein. The renal district of the IVC originates after the right and left subcardinal venous anastomoses coalesce. This complex process may lead to abnormal persistence or regression of some fetal structures causing IVC anomalies.

Agenesis IVC (AIVC) consisted of three major configurations which are the following: (1) the absence of the suprarenal IVC resulting from the right subcardinal vein failure; (2) the hepatic segment draining directly in the right atrium; (3) the azygos and hemyazigos systems providing the drainage from the infrarenal IVC. This kind of anomaly is associated with other multiorgan defects, such as poly- and asplenia, Kartagener syndrome, and heart defects. The following findings could be further drawn: the absence of the infrarenal IVC with preservation of renal and suprarenal segments is related to a failure of the right supracardinal vein; the complete absence of the IVC is linked to the failure of all the three paired vein networks.

The causes of these conditions are not fully understood. Nevertheless, embryonic disontogenesis and intrauterine or perinatal thrombosis are two of the main causative hypotheses.

The literature revealed that IVC anomalies may be associated with renal malformations, involving the right kidney (agenesis, hypoplasia, and aplasia). For example, the triad of kidney and IVC anomaly and extensive venous thrombosis both represent a rare condition called KILT syndrome (2).

Agenesis IVC could be asymptomatic, and the diagnosis could be made accidentally during other diagnostic evaluations. DVT is the most common presentation. The inadequate capacity of the collateral venous system leads to venous blood stasis facilitating the formation of blood clots.

Generally, patients with AIVC are younger with no other risk factors for DVT. The iliac vein is the most common location of thrombosis with a higher frequency of bilateral involvement and higher DVT recurrence. Pulmonary embolism is a rare manifestation, blood clots are blocked into aberrant collateral vessels (3). Recurrent DVT events and venous stasis could also lead to chronic venous insufficiency with ulceration and the appearance of trophic lesions (4).

The absence of a variable segment of the IVC leads to the development of extensive collateral circuits able to convey venous blood flow from the lower part of the body toward the right atrium. As seen in the case presented, azygos and hemiazygos veins are the two main collectors of venous blood flow. In their thoracic district, they reached similar diameters to the aorta thanks to their high capacitance. Ascending lumbar veins, paravertebral plexus, and anterior abdominal wall veins are also included in the numerous collateralization systems involved in this kind of anatomical malformation.

Duplex vascular ultrasound is usually the first diagnostic exam to be done, even if anatomical anomalies are not always diagnosed. In case of diagnostic doubts, CT-angiography and MRI are the most informative examinations.

Anticoagulation is the mainstay of the treatment in these kinds of patients. The optimal duration is not well established. Indefinite anticoagulation is sometimes advocated due to the high risk of DVT recurrence even if a close risk of bleeding monitoring could be advised (5). Results from the RIETE Registry outlined interesting data about 31 patients with AIVC treated with anticoagulation agents, with a mean duration of 3 years. One year after the first event of DVT, these patients had a higher rate of lower limb skin induration, collateral vein circulation, or venous ulcer respect to patients without AIVC (6).

A recent article confirms the importance of long-term anticoagulation. Briefly, in a series of 9 patients with AIVC, only two patients experienced DVT re-occurrence during a mean follow-up of nearly 78 months, in which the anticoagulation regimen was withdrawn in these two patients (7).

On the other hand, other published data reveal that the risk of DVT recurrence after stopping anticoagulation therapy remains low (8). As reported, the literature remains ambiguous in terms of type and duration of anticoagulation therapy considering the risk of DVT recurrence and the risk of hemorrhagic events. The ambiguity is maintained regarding the rationale of the eventual interventional approach. Thrombolysis is advocated as a possible therapeutic approach of DVT in AIVC patients as suggested by some authors (9) with conflicting results. Catheter-directed thrombolysis has been related to rapid symptoms relief and removal of thrombotic burden, leading to a reduction of possible DVT re-occurrence. We decided to not undergo this path because we were not sure about the real-time onset of thrombosis even if the patient referred that symptom started a week before. In addition, due to the chronic obstructive condition of the IVC, we would expect a significant rate of recurrence of DVT, even if initial thrombolysis was successful. Lastly, the patient was initially admitted to a peripheral hospital with limited facilities. On the other side, open surgery reconstruction seems to be effective and appears to prevent the deterioration of chronic vein insufficiency over time, as highlighted by Sagban et al. (10).

## Conclusion

Physicians need to consider the eventuality of IVC anomalies in a young adult presenting with unexplained, extensive, or bilateral DVT. Accurate diagnostic evaluation is necessary to fully identify this condition that could represent a real challenge.

Conservative treatment with long-term anticoagulation therapy or interventional approach represents two different modalities of treatment although literature lacks clear indications due to the rarity of the condition. In this perspective, further studies with large cohorts are needed to standardize the management of this particular condition.

## Data Availability Statement

The original contributions presented in the study are included in the article/supplementary material, further inquiries can be directed to the corresponding author.

## Ethics Statement

Written informed consent was obtained from the patient for publication. The Ethical Committee of the hospital was informed of the no-experimental design of the study.

## Patient'S Perspective

Young adults presenting with a complex scenario of deep vein thrombosis are challenging situations. The prompt and correct identification of the underlying cause is mandatory to plan the best treatment. In this case, the identification of the IVC malformation has been critical for the correct management, especially in the medium and long-term perspectives. In this light, both young patients and physicians must be aware of this remote but important condition that could be related to critical complications. Unfortunately, this kind of diagnosis has a significant impact on the life of patients in terms of long-term anticoagulant therapy, mandatory and strict control of risk factors, and the possible occurring of new events of deep vein thrombosis.

## Author Contributions

EP and GD: conception and design, writing, critical revision, approval of the manuscript, and general supervision. SC, RM, VP, and IC: data collection, critical revision, and approval of the manuscript. LA and GP: critical revision, approval of the manuscript, and general supervision. All authors contributed to the article and approved the submitted version.

## Conflict of Interest

The authors declare that the research was conducted in the absence of any commercial or financial relationships that could be construed as a potential conflict of interest.

## Publisher's Note

All claims expressed in this article are solely those of the authors and do not necessarily represent those of their affiliated organizations, or those of the publisher, the editors and the reviewers. Any product that may be evaluated in this article, or claim that may be made by its manufacturer, is not guaranteed or endorsed by the publisher.

## Abbreviations

IVC: Inferior Vena Cava
AIVC: Agenesis Inferior Vena Cava
DVT: Deep Vein Thrombosis.
